# When Children's Needs Do Not Fit the System: Street‐Level Bureaucrats Facilitating Home Care for Children Living With Respiratory Support

**DOI:** 10.1111/nin.70175

**Published:** 2026-10-13

**Authors:** Johan Florén, Berit Lindahl, Sepideh Olausson, Agneta Markström, Andreas Palm, Åsa Israelsson‐Skogsberg

**Affiliations:** ^1^ Department of Caring Science, Faculty of Caring Science, Work Life and Social Welfare University of Borås Borås Sweden; ^2^ Institute of Health and Care Sciences, Diagnostics, Acute and Critical Care University of Gothenburg Gothenburg Sweden; ^3^ Department of Medical Sciences, Respiratory, Allergy and Sleep Research Uppsala University Uppsala Sweden; ^4^ Cardiothoracic Intensive Care Unit (TIVA), Sahlgrenska University Hospital Gothenburg Sweden

**Keywords:** child, delivery of healthcare, health equity, health services accessibility, respiratory therapy

## Abstract

This study explores the meanings of acting as a street‐level bureaucrat in facilitating home care for children living with respiratory support and their families. A phenomenological hermeneutic approach was used, based on interviews with professionals involved in assessment, decision‐making and care provision across different parts of the welfare system. The findings show that this work is lived in tensions between compassion and regulation, closeness and distance, and enduring needs and finite resources. Acting as a bureaucrat in this context means being confronted by the vulnerability of the child and family while being bound to formal language, legal categories and organisational limits. Home care is made possible through these structures, yet never fully contained by them. Responsibility emerges as exposed, stretching beyond the moment of decision and continuing into a life that unfolds elsewhere. Bureaucratic work thus appears as more than administration. It is lived as an effort to respond to what is pressing and fragile while remaining within frameworks that cannot fully receive it. The study highlights the importance of institutions that not only uphold justice but also make good care possible.

## 1 Introduction

Care is shaped not only through direct encounters between individuals and professionals but also through administrative, legal, economic and organisational processes that determine which needs are recognised, what support is granted and how care can be sustained over time. Decisions concerning eligibility, resources, organisation and responsibility may therefore have direct consequences for people's everyday lives. Understanding care requires attention not only to those who provide it in direct encounters but also to the professionals who translate welfare policy into individual decisions and practical arrangements.

Street‐level bureaucrats are professionals who work at the interface between public policy and people's everyday lives (Lipsky 2010). They interpret and apply laws, regulations and organisational guidelines in individual cases, often under conditions of limited resources, incomplete information, competing demands and organisational pressure. Because general rules cannot anticipate the full complexity of individual circumstances, their work necessarily involves interpretation and discretion. Street‐level bureaucrats therefore do not merely implement policy. Through their assessments, priorities and decisions, they participate in shaping how policy is enacted and experienced in practice. Street‐level bureaucracy has been widely used to examine how professionals interpret policy and exercise discretion when general rules are applied to individual situations (Chang and Brewer 2023; Evans 2004). Recent studies further show its relevance for understanding policy implementation and how access to healthcare and social services is shaped in practice (Litchfield et al. 2023; Rossi et al. 2025).

Ray's theory of Bureaucratic Caring offers a caring science perspective on these institutional processes. The theory emerged from research on caring within complex healthcare organisations and challenges a simple opposition between caring and bureaucracy (Ray 1989, 2021). Instead, caring is understood as embedded within the ethical, spiritual, legal, political, economic, educational, technological and organisational dimensions of institutional life. These dimensions are interrelated and shape how care can be understood and enacted. Bureaucratic structures may support care through coordination, continuity, accountability and access to resources, while also restricting it through standardised categories, procedural requirements, organisational boundaries and economic priorities. Administrative and organisational decisions are therefore not external to care, but form part of the conditions through which care becomes possible, limited, justified and sustained.

Lipsky's concept of street‐level bureaucrats directs attention to the position, discretion and everyday practices of professionals working between policy and lived experience. Ray's theory, in turn, illuminates the caring and ethical significance of the institutional structures within which this work occurs. Together, these perspectives allow street‐level bureaucratic work to be understood as more than the implementation of regulations. It is also part of the process through which human needs are translated into formal categories and through which care is recognised, organised, resourced and brought into everyday life.

These issues become particularly consequential when administrative and organisational decisions concern children and young people who depend on long‐term respiratory support at home. Such support may include mechanical ventilation, continuous positive airway pressure, tracheostomy or high‐flow oxygen. These children often have complex and long‐lasting care needs requiring skilled interventions, monitoring and extensive support from families and professionals (Amaddeo et al. 2020; Falkson et al. 2017; Graham et al. 2017; Popejoy et al. 2021). Advances in respiratory technology and medical care have enabled children with previously life‐threatening conditions to survive and remain part of family life, education and society (Castro Codesal et al. 2017; Falkson et al. 2017; Israelsson‐Skogsberg et al. 2018; Kwak 2023; Russell et al. 2024). Yet living at home requires more than access to respiratory technology. Children and young people strive for participation, independence and an everyday life centred on school, friendships, leisure activities and future aspirations (Israelsson‐Skogsberg et al. 2018, 2023). Realising these possibilities may depend on reliable care arrangements, competent caregivers and continuous or recurrent monitoring to ensure safety and respond to changing needs (Falkson et al. 2017; Israelsson‐Skogsberg et al. 2018; Ledin et al. 2024). Parents often carry substantial responsibility for coordinating care, maintaining continuity and compensating when formal support is insufficient (Bjerregaard Alrø et al. 2021; Falkson et al. 2017; Ledin et al. 2024). Decisions about formal support may therefore shape not only access to services but also the child's safety, participation, family relationships and ability to continue living at home.

In Sweden, these arrangements are situated within a welfare system involving healthcare, municipal social services and the social insurance system. The Act concerning Support and Service for Persons with Certain Functional Impairments (LSS) aims to secure good living conditions through individually tailored support, including personal care assistance, which represents the most comprehensive form of home‐based care and support (Clevnert and Johansson 2007; Rauch et al. 2018; SFS 1993:387 1993; Swedish Social Insurance Agency 2024). Families may apply for personal care assistance through the Swedish Social Insurance Agency or their local municipality. Eligibility is based on legally defined criteria, including an assessment of basic needs. When a child does not meet the criteria for state‐funded personal care assistance, the municipality remains responsible for assessing the need for support under the applicable legislation (Figure 1).

**Figure 1:**
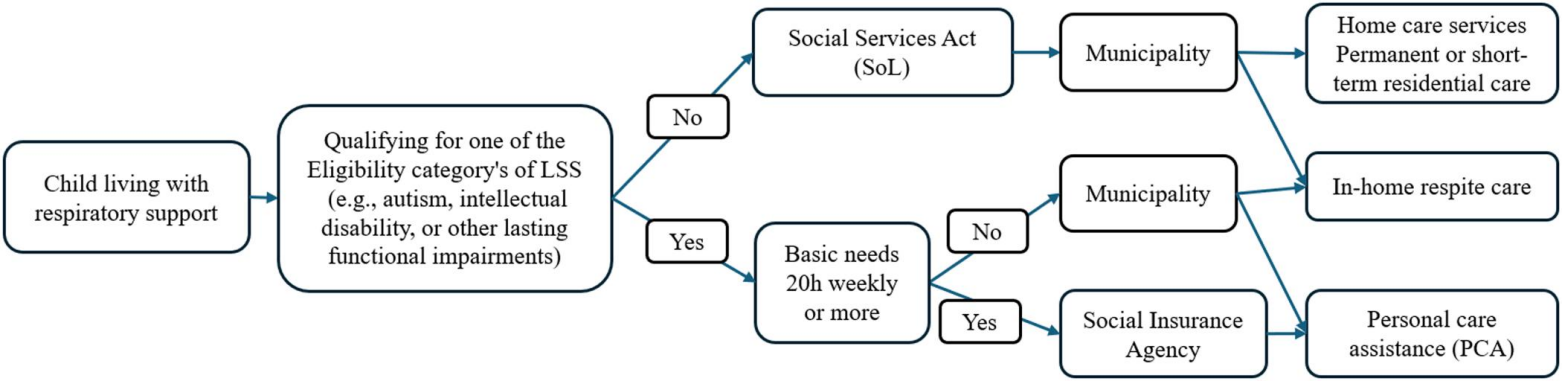
Flowchart displaying the process of applying for long‐term home care for a child living with respiratory support.

For children living with respiratory support, personal care assistance has been described as crucial and, in some cases, a prerequisite for safety, participation and continued family life at home (Alrø et al. 2021; Israelsson‐Skogsberg et al. 2018). Access to assistance is nevertheless mediated through administrative and legal processes that families may experience as complex, time‐consuming and difficult to navigate. These processes can place substantial demands on parents to document, explain and advocate for the child's needs (Egard and Hedvall 2021; Israelsson‐Skogsberg et al. 2023; Olin and Dunér 2019). Between 2015 and 2021, 29% of 600 children living with respiratory support in Sweden were granted personal care assistance (Florén et al. 2025).

The legal conditions governing personal care assistance for children living with respiratory support have changed substantially. A shift in legal practice in 2015 excluded breathing as grounds for personal care assistance unless it was associated with mental impairment. This contributed to some families losing access to personal care assistance and instead being referred to municipal social services (Berggren et al. 2021; Brennan et al. 2016; HFD 2015:46 2015; Rauch et al. 2018). In 2019, breathing was re‐established as a basic need, allowing assistance related to activities such as tracheostomy care and secretion mobilisation to be considered in eligibility assessments (SFS 1993:387 1993; Swedish Government Offices 2019). Although the reform was followed by increased approval rates and allocated hours, families have continued to report uncertainty concerning reassessments, continuity and the long‐term stability of care arrangements (Egard and Hedvall 2021; Florén et al. 2025; Israelsson‐Skogsberg et al. 2023; Swedish Social Insurance Agency 2021).

Within this system, street‐level bureaucrats occupy a consequential position. They must interpret legal and organisational frameworks, assess whether complex and sometimes life‐sustaining needs correspond to formal criteria, allocate finite resources and organise services that must function safely and continuously in everyday life. Their work takes place between the singular and changing realities of children and families and the general categories, procedures and economic conditions of the welfare system. Decisions made within these processes may determine not only whether support is granted but also how care is organised, who is expected to carry responsibility and whether the arrangement can be sustained over time. In the present study, street‐level bureaucrats include caseworkers responsible for assessing eligibility within municipalities, social services and the Swedish Social Insurance Agency, as well as managers and legal support professionals involved in the organisation, legal framing and provision of services. Their organisational positions and responsibilities differ. However, all are involved in interpreting and applying legal, organisational and economic frameworks in relation to the needs and expressed wishes of children and families. In this sense, they participate in translating welfare policy into the practical conditions of home life. Viewed through Bureaucratic Caring, they are therefore not external to care, but part of the institutional practices through which care takes form.

While the experiences of children and families living with respiratory support have been described in previous research (Alrø et al. 2021; Bjerregaard Alrø et al. 2021; Israelsson‐Skogsberg et al. 2018, 2019, 2023; Ledin et al. 2024), less attention has been paid to the perspectives of the street‐level bureaucrats involved in making such care possible. Given the emotional, practical and organisational complexity of this work, deeper understanding is needed of how street‐level bureaucrats experience and navigate this landscape. This study therefore aimed to explore the meanings of acting as a street‐level bureaucrat in facilitating home care for children living with respiratory support and their families.

## 2 Method

The present study employs a phenomenological–hermeneutic approach influenced by the work of the French philosopher Ricoeur (1976), and further developed through the methodological contributions of Lindseth and Norberg (2004, 2022). Grounded in a lifeworld perspective, this approach seeks to describe, deepen understanding of and explore the meanings of lived experience. In the present study, it is used to illuminate the meanings of acting as a street‐level bureaucrat in facilitating home care for children living with respiratory support and their families. Similar methodological frameworks have previously been applied in research focusing on children living with respiratory support and their families (Bjerregaard Alrø et al. 2021), as well as in studies of professionals' lived experiences (Alrø et al. 2022).

### 2.1 Participants and Settings

A purposive sampling strategy (Patton 2015) was used to capture a broad range of professional perspectives from different sectors of the welfare system. The sampling represented maximal variation (Polit and Beck 2021), that is, purposive in that professionals were deliberately sought from different organisations, occupational roles, geographical areas and stages of the process through which home care is assessed, organised and implemented. Participants included caseworkers responsible for assessing eligibility for home care within municipalities, the Social Insurance Agency and social services. All had experience of handling cases involving children living with respiratory support. The study also included line managers and legal support professionals involved in providing home care for children living with respiratory support, to capture the full chain from assessment and decision‐making to implementation. Across their different roles, all participants were tasked with interpreting and applying legal and economic frameworks in relation to the needs and expressed wishes of children and families. All participants held roles that positioned them as street‐level bureaucrats in the sense described by Lipsky (2010), and are henceforth referred to as bureaucrats or participants.

The inclusion criteria required participants to be professionals responsible for assessing, coordinating or managing the delivery of home care to children living with respiratory support. The participants (*n* = 17) were aged between 24 and 64 years, with a median age of 41 years. Four participants were men, and 13 were women. Their educational backgrounds varied in both field and level, although social work was the most common professional qualification. Other backgrounds included university education in law, public law, human rights, teaching, nursing and healthcare management as well as vocational education in disability support, assistant nursing and support pedagogy.

Participants were recruited via email or telephone to their respective organisations, requesting individuals with experience of working with children living with respiratory support in their current role. Nationwide coverage was achieved, with participants representing regions from southern to northern Sweden, including both large urban (> 200,000 inhabitants) municipalities and smaller rural ones (< 40,000 inhabitants) (Swedish Association of Local Authorities and Regions 2023) (Table 1).

**Table 1:** Characteristics listing occupation, age, organisation and area of operations for all participants in the study.

Occupation	Age	Organisation	Area
Case manager LSS	40	Municipality	Mid‐sized municipality
Case manager LSS	47	Social Insurance Agency	Large city
Case manager LSS	63	Municipality	Small municipality
Case manager LSS	39	Social Insurance Agency	Large city
Case manager LSS	32	Municipality	Large city
Case manager LSS	24	Municipality	Mid‐sized municipality
Case manager LSS/social services	32	Municipality	Mid‐sized municipality
Case manager LSS	49	Municipality	Mid‐sized municipality
Case manager LSS	36	Municipality	Large city
Line manager of permanent accommodation	54	Municipality	Large city
Line manager of PCA	42	Municipality	Mid‐sized municipality
Line manager of PCA	43	Municipality	Small municipality
Line manager of short‐term accommodation	39	Municipality	Mid‐sized municipality
Line manager of short‐term accommodation	57	Municipality	Mid‐sized municipality
Line manager of PCA		Private PCA provider	Large city
Line manager of PCA	37	Private PCA provider	Large city
Juridical support	54	Private PCA provider	Large city

*Note:* LSS case manager: Investigates cases and prepares recommendations for decisions on care under LSS. Line manager: Responsible for staff, budget and day‐to‐day operations within PCA services or permanent or short‐term accommodation. Legal support officer: Provides legal support in matters concerning LSS and in the application and reassessment process.

### 2.2 Data Collection

Data were collected through semi‐structured interviews (Brinkmann and Kvale 2015). All interviews were conducted in Swedish by the first author between October 2025 and February 2026. Interview duration ranged from 21 to 45 minutes (median 37 minutes). One interview was conducted in person, one by telephone because of technical difficulties and the remaining interviews were conducted digitally via Microsoft Teams, as participants were well accustomed to digital meetings and used Microsoft Teams daily in their work (Thunberg and Arnell 2022). Participants were either at their workplace or working remotely from home. All interviews were audiorecorded and transcribed using Amberscript (Amberscript.com), followed by relistening and correction of the text. The quotations presented in the article were translated from Swedish into English by the authors, with attention to preserving their meaning and tone.

Each interview began with the introductory question: ‘Can you please tell me about your role in situations where you encounter children living with respiratory support’. This prompted dialogue in which participants described their experiences of working with children living with respiratory support, particularly in relation to care and PCA provision. Follow‐up questions such as ‘Tell me about a situation that affected you’ or ‘How did this affect you?’ were asked to stimulate reflection.

### 2.3 Data Analysis

The analysis followed the phenomenological hermeneutical method described by Lindseth and Norberg (2004, 2022), inspired by Ricoeur's (1976) theory of interpretation. The process was dialectical rather than linear, involving continuous movement between the whole and its parts (Figure 2). The first phase consisted of a naïve reading of the interview transcripts. The text was read several times to grasp its meaning as a whole and allow an initial, tentative understanding of the phenomenon to emerge: a naïve understanding. This naïve understanding offered a preliminary interpretation of the phenomenon as a whole and guided the subsequent structural analysis. The structural analysis began with close attention to the language used by participants. Expressions, formulations and recurring concepts were identified as key terms in an initial analytical step, acknowledging that meaning is carried and shaped through language. After the identification of shared concepts and terms, the analysis proceeded to a second structural step aimed at organising the text into larger units of understanding. Meaning units were then delineated, condensed and grouped into subthemes and themes with the support of NVivo. In this explanatory phase, the aim was to describe what the text says by clarifying patterns of meaning. The structural analysis functioned as a critical validation and examination of the naïve understanding. When tensions or inconsistencies appeared, the initial understanding was reconsidered and reformulated. Interpretation thus evolved through a back‐and‐forth movement between an overall grasp and detailed analysis of the parts.

**Figure 2:**
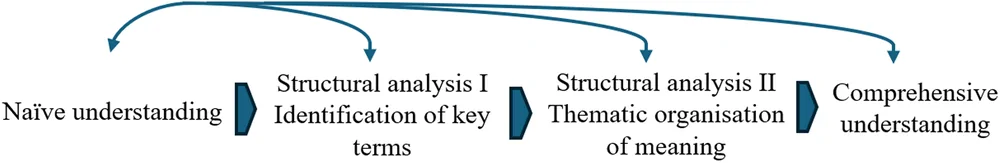
Description of the analytical process, moving back and forth from naïve understanding to structural analysis and comprehensive understanding.

In the final phase, a comprehensive understanding was formulated. The naïve understanding and the validated themes were brought together into an interpreted whole. This interpretation was further deepened through reflection on the researchers' preunderstandings and in dialogue with relevant theoretical perspectives. The understanding represents one, and the most plausible, interpretation of the phenomenon, grounded in the text and shaped within a particular horizon of understanding.

Rigour was sought in accordance with the principles outlined by Lindseth and Norberg (2004, 2022). All authors participated in repeated readings of the material and continuous interpretive dialogue throughout the analytic process. Interpretations were systematically questioned and tested against the text to avoid premature closure and ensure coherence between parts and whole. The research group consists of physicians and nurses experienced in the method, and the interpretation was therefore shaped within caring science perspectives. The caring science background supported sensitivity to issues of vulnerability, responsibility and care, while also requiring ongoing reflexive awareness of how preunderstandings might influence interpretation. An audit trail, including analytic notes, thematic development and reflections on preunderstanding, was maintained to enhance transparency and credibility (Morse et al. 2002).

### 2.4 Ethical Considerations

The study was conducted in accordance with the Declaration of Helsinki (World Medical Association 2024) and was approved by the Swedish Regional Ethical Review Board (Dnr 2025 01875 01). Participants received informed consent before the interviews. The informed consent emphasised that participation was voluntary and that participants had the right to withdraw at any time without giving a reason. This information was also repeated verbally at the start of each interview.

## 3 Results

The first part presents the naïve understanding: the initial understanding of the whole and a qualified guess that initiated the analytical process.

### 3.1 Naïve Understanding

Meeting children living with respiratory support means being emotionally affected. The role of the bureaucrat presents itself through the use of formal and distanced language. Being a bureaucrat means presenting oneself as part of a collective. The bureaucrat moves between the desire to grant care and the constraints of legislation, searching for justification in pivotal certificates written from another professional perspective and in another professional language, while simultaneously safeguarding the system from misuse. This creates a tension between ‘the head and the heart’. A need for reassurance and a felt obligation to do what is right present themselves to the participants. This need for formal principles means adapting to the map of rules rather than the territory of lived reality. A sense of urgency is connected to the process.

Being a bureaucrat means being confronted with financial constraints that govern the recruitment of personal care assistants, despite knowing that the competency for which one is responsible is sometimes lacking. This responsibility is exercised at a distance and judged by the family.

The naïve reading pointed to a tension in which the bureaucrat appeared emotionally affected yet bound to speak and act with formal restraint. This tension was carried through a distinct and formalised language. Language therefore became an important point of entry for the continued analysis, and the first structural analysis focused on the concepts and terms used in the interviews.

### 3.2 Structural Analysis I: Identification of Concepts and Terms

This phase constituted the first structured step in the analysis and was conducted with a critical and explanatory attitude towards the text. Its purpose was to examine and validate the naïve understanding. A distinct use of language emerged in the interviews, shaped by legal and administrative reasoning rather than everyday speech. Language guided the process by delimiting what could be expressed, justified and understood. The analysis therefore focused on concepts and recurring expressions, and on the meanings they were given within the interview context. Key terms were identified and interpreted in relation to how they were articulated and described by the participants (Table 2).

**Table 2:** Key terms and their meanings in the context of providing care to children living with respiratory support, with associated interview excerpts.

*Eligibility category*
Meaning: A legal category defining who qualifies for care under LSS, and thereby whether the individual falls within the scope of the legislation. *Quote: You could argue it either way, and assistance is very particular in that you often either qualify for a fairly extensive decision or for nothing at all.*
*Basic needs*
Meaning: Core personal needs that may entitle an individual to PCA, such as hygiene, meals, dressing, communication and breathing. *Quote: Around seven hours to be granted personal assistance by the municipality. And then you look at the basic needs, such as personal hygiene, dressing and undressing, meals, communication, preventive support, breathing support, and medical support*.
*Permanence*
Meaning: The expected duration of the condition and care needs. To be eligible for PCA, the condition or care need must be long‐term, not temporary. *Quote: It is the eligibility category assessment that is difficult when it comes to children, because you need to prove the long‐term nature of the condition*.
*Fear of life*
Meaning: An immediate risk of life‐threatening consequences if continuous supervision is not provided. Long‐term consequences are not sufficient under LSS.
*Quote: So if there is a risk to life, it can count as a basic need. But supervision, if there is no risk to life, and intermittent interventions when there is no risk to life, may instead be classified as other needs, and that presupposes that assistance has already been granted on other grounds*.
*Decision‐maker*
Meaning: The person or authority formally responsible for making the decision, such as a senior case manager, manager or political board, depending on the organisation or municipality. *Quote: Yes, we have divided it so that one person carries out the investigation itself, and another always quality assures it to make sure the decisions do not become arbitrary and that they comply with the legislation*.
*Parental deduction*
Meaning: A reduction of hours in cases involving children, based on what parents are expected to do within ordinary parental responsibility. *Quote: Before 2023, there were many disputes about the extent of parental responsibility and what actually counted as parental responsibility. This had been litigated for years. There were never any proper investigations showing what children normally need help with, so the municipality and the Social Insurance Agency had to make rough estimates. Then a standardised model was introduced in 2023…*
*Supporting documentation*
Meaning: All documents used for the decision: medical certificates, assessments and statements.
*Quote: There must always be objective information confirming what the parents say. It is not enough for a parent to say that their child needs help with breathing; there must be a medical certificate*.
*Reassessment*
Meaning: When the authority re‐evaluates an existing decision, either regularly or due to changed circumstances. *Quote: After we have made the decision, we usually reassess it after two years*.
*Current legal position*
Meaning: The prevailing legal interpretation of the law, often shaped by previous court decisions and administrative practice. *Quote: Before that legislative change came into force, I thought quite a few would qualify on those grounds. But then the first court rulings came, and the interpretation was very narrow, which meant that very few actually qualified*.
*Legal certainty*
Meaning: The principle that decisions must be lawful, predictable and procedurally fair. *Quote: My role at present is mainly supervisory in the way I become involved in these cases, so it is about ensuring legal certainty, relating to case law, making sure things are handled correctly, and that the right scope of support is granted*.
*Appeal*
Meaning: When the individual challenges a decision and asks a higher authority or court to review it. *Quote: They rarely change their decisions themselves… which is why the Social Insurance Agency has a reconsideration unit that reviews everything. But even there… In court, however, decisions are changed more often than they are internally. That makes a big difference, and many cases end up in court…*

### 3.3 Structural Analysis II: Thematic Organisation of Meaning

Following the identification of shared key terms, the second step of the structural analysis addressed how these meanings were enacted. The analysis examined how participants experience the bureaucrat role in facilitating home care for children living with respiratory support and their families. The interview transcripts were reread several times. Meaning units were identified and condensed. The condensed meaning units were then sorted according to similarities and differences to organise the data before the development of subthemes and themes (Table 3). The themes and subthemes are presented in Table 4.

**Table 3:** Example of the analytic steps in the structural analysis.

Meaning unit	Condensed meaning unit	Subtheme	Theme
There must always be objective information confirming what the parents say. It is not enough for a parent to say that their child needs help with breathing; there must also be a medical certificate	The information must be objective and confirm parents' statements. A medical statement needs to verify/confirm the child's needs	Reshape the narratives to fit the language of the system	Eligibility as living the tension between compassion and regulation
To see a child who wants to be like everyone else, and then to be the one who may not make that possible because the need has not been substantiated or does not amount to sufficient time, that is something we can definitely take home with us	Not being able to meet the child's need can stay with you	Feelings of compassion	

**Table 4:** Overview of themes and corresponding subthemes.

Subtheme	Theme
Feelings of compassion Reshape the narratives to fit the language of the system	Eligibility means living the tension between compassion and regulation
Distancing as a way of relating within responsibility	
Responsibility continues beyond the decision Resources surrounding are measured and finite	Responsibility means being stretched between finite resources and enduring needs

#### 3.3.1 Eligibility Means Living the Tension Between Compassion and Regulation

Assessment takes shape in the movement between responsiveness to vulnerability and the institutional forms through which reality must be rendered recognisable, while distance becomes part of how responsibility is carried and diffused.

##### 3.3.1.1 Feelings of Compassion

Acting as a bureaucrat in facilitating home care for children living with respiratory support means dwelling within a field of competing meanings. At its core lies compassion. To stand before a vulnerable child and exhausted parents means being confronted by a situation of profound dependence. Their exposure reaches beyond documents and procedures. The child's reliance on constant care, and the parents' inability to leave even briefly, make their situation difficult to hold at a distance. The encounter is not only a case to be assessed, but a life situation that calls for response. In this, a wish to help takes shape as a movement towards the other, and responsibility emerges as a call that is difficult to refuse.

Children should not have to depend on their parents in that way, and parents should be able to be parents. Some children may die if the parent leaves the room to go to the toilet.

(IP2)

A sense of pride may arise when one does more than what is strictly required. It is rooted in taking initiative, contacting physicians directly, handling matters with urgency and ensuring that no essential detail is overlooked. In such moments, professional responsibility comes to exceed routine procedure. Yet this initiative coexists with a persistent demand for objectivity, adherence to established procedures and the safeguarding of impartiality and legal certainty.

I often do not need certificates. Instead of relying on a parent's account, I call the responsible physician directly, ask my questions, and document the conversation in the assessment.

(IP12)

##### 3.3.1.2 Reshape the Narratives to Fit the Language of the System

Compassion towards the child and family proves inseparable from loyalty to the regulatory framework. Acting responsibly means adhering to rules, certificates and predefined criteria that determine eligibility.

Detailed certificates are the most important thing, because nothing can be granted unless it is substantiated.

(IP1)

The rules governing the provision of care stand apart from personal moral impulses. They are impersonal, binary and at times meticulous to the point of rigidity. Within this structure, frustration may arise, as complex and fragile life situations must be translated into categorical distinctions. The child's and family's situation does not appear merely as an administrative matter, but as something that confronts the assessor as an ethical demand.

We can see that parents often take on a very large share of the responsibility in these cases, which we are not always fully satisfied with. But from the authority's perspective, we still consider the assessment to be correct. What one feels emotionally may be something else.

(IP10)

Responding to this ethical call means searching formal documents for what can make the child's situation visible within the system: life‐threatening conditions, duration and eligibility within the relevant legal category. Evidence is sought in medical certificates that are often difficult to interpret and written from another professional horizon. The child's needs must be articulated through specific formulations to become recognisable within the system. Yet this does not make the assessment straightforward. Objectivity does not present itself as readily given, but as something that must be upheld amid uncertainty. Acting means not only translating the child's situation into administratively recognisable terms, but also sustaining judgement in the tension between different professional horizons.

It can be difficult to ask concrete questions, because healthcare may at times respond in ways that favour the individual, while we are still expected to maintain an objective assessment.

(IP10)

##### 3.3.1.3 Distancing as a Way of Relating Within Responsibility

Being a bureaucrat requires distancing. A formal and restrained language comes into use. Encounters with the child may be regarded as unnecessary, particularly when the child is unable to verbalise their will. Denials are justified in terms of what the rules allow, and through this regulatory language responsibility is shared with decision‐makers and the wider system. Such distancing may be understood as a way of not having to carry the ethical burden alone. At the same time, responsibility does not disappear into the system. It may remain with the bureaucrat as something personally felt and difficult to step out of. In this, distance does not stand outside responsibility, but becomes one way in which it is carried.

I do not always need to meet the child. If the medical documentation is sufficient, seeing a child connected to equipment serves little purpose. But the certificates can still affect you deeply, because they make visible just how seriously ill the child is.

(IP6)

Within this tension between compassion and regulation, distancing as a way of relating within responsibility is also shaped by how earlier legal frameworks remain present in experience. For those with longer experience, previous rulings remain in the background as more than historical knowledge; they form a lived horizon against which current decisions are understood. Being a bureaucrat is thus shaped not only in relation to the case at hand but also through the remembered experience of a time when extensive needs could remain unmet within the limits of the law. For others, this responsibility takes shape with less to lean on. When similar cases are few and shared understandings remain uncertain, responsibility may be carried in solitude. It is then not only distributed through rules and structures but also remains close to the one who must judge.

It feels much more positive now that breathing has been recognised as a basic need. There was a period when the legal interpretation was so restrictive that, despite very extensive support needs, there was nothing we could do.

(IP2)

#### 3.3.2 Responsibility Means Being Stretched Between Finite Resources and Enduring Needs

Responsibility does not come to rest with the decision itself but continues into a reality where needs endure while resources remain limited. Acting means granting, delimiting or handing over care within finite organisational conditions, while knowing that the child's and family's life continues beyond what the system can fully provide.

##### 3.3.2.1 Responsibility Continues Beyond the Decision

When eligibility has been established, something settles and something begins. Granting care carries a feeling of release, as if a weight briefly lifts. The responsibility that has pressed during the assessment finds a temporary point of rest. There is often a sense that what could be given within the limits of the system has been given. The everyday life of the family continues beyond the immediate sphere of decision‐making, and the consequences of the decision unfold elsewhere. What was once intensely present recedes from view. Responsibility remains, but without proximity.

I sit in my little bubble here. I make the decision, and then it goes out into the world. I do not follow these children afterwards, because they eventually move on to the Social Insurance Agency.

(IP6)

When care is not granted, the decision does not come to rest. It lingers. The reasoning remains open to further examination, and professional judgement may come to feel exposed. What had appeared justified and concluded may be unsettled when the case is reassessed in court. In this, certainty may begin to waver.

After many rounds, we concluded that assistance could not be granted because the physician expected the need to end within a few months. But the case went to court, and the court disagreed, so the child was granted assistance.

(IP4)

At the same time, there is reassurance in knowing that denial does not necessarily mean that care will be absent. Since this is not the final authority, other actors, such as social services, may intervene and this may bring some relief in the face of denial.

We are not the final authority, since social services have lower thresholds than we do. It is a relief to know that it does not depend on me alone.

(IP4)

##### 3.3.2.2 Resources Surrounding Are Measured and Finite

As the decision takes form in real life, responsibility changes character. What was once words on paper becomes practice, night shifts and breathing. There is pride in seeing care take shape, in knowing that something complex is being carried. At the same time, an underlying unease accompanies this realisation, as advanced care comes to depend on continuity, presence and competence in everyday practice. The fragility of this arrangement can be felt as a background tension, a subtle stress tied to remaining responsible for something that requires ongoing follow‐up but cannot be fully overseen at all times.

The assistance sector is marked by staff turnover, short notice periods, and rapid recruitment. These cases therefore place high demands on managers to be present, follow up closely, and have sufficient competence to support their staff.

(IP7)

Acting as a bureaucrat means working within a persistent tension between extensive needs and limited resources. Economic constraints are not experienced as abstract conditions, but as factors that enter everyday judgements about what can be granted, extended or sustained. The child's needs appear continuous and far‐reaching, while the resources available are measured and finite. In this gap, facilitating care means not fully providing what is needed, but trying to make arrangements within what the system can bear. This may evoke a sense of insufficiency, as if the care made possible never entirely matches the complexity of the life it is intended to sustain.

No, it does not cover the costs. We have repeatedly applied to the Social Insurance Agency for a higher hourly rate because this is such a complex case, but the requests have been denied.

(IP15)

Within this understanding of facilitating care, parents become crucial to making care workable. Their presence may bring relief, especially when they step in where formal coverage falls short. At the same time, this reveals that home care is upheld not only by formal care alone but also by the ongoing effort, flexibility and endurance of the family. When parents express trust and describe feeling safe, the professional's efforts are affirmed. When worry remains, however, the fragility of the arrangement becomes visible once more. Acting as a bureaucrat thus involves not only enabling care, but doing so in the awareness that what is granted may still depend on parents carrying more than formal care provides.

Parents may hope that assistants will take over much of the care, but that never fully happens. They still have to step in a great deal, not least because assistants are often absent due to illness.

(IP3)

Within this quiet oscillation between relief and unease, pride and insufficiency, distance and proximity, the meaning of facilitating home care takes shape. It is experienced as standing between measured resources and immeasurable needs, holding a responsibility that never fully settles.

### 3.4 Comprehensive Understanding

As a final step, a comprehensive understanding of the whole was formulated through an interpretive synthesis of the naïve reading, the structural analyses, the researchers' theoretical preunderstanding and relevant literature. The text shows that acting as a bureaucrat in facilitating home care for children living with respiratory support means carrying an exposed responsibility within structural limits. Whether assessing eligibility or sensing how decisions continue into the child's and family's everyday life, professional being is formed in the tension between vulnerability and regulation, and between enduring needs and finite resources. Acting means responding to what appears pressing and fragile while remaining bound to frameworks that cannot fully receive it. Meaning emerges in the space between compassion and law, economy and lived life. Bureaucratic work thus appears not merely as administrative, but as a way of being stretched between what is called for and what is possible.

In Lipsky (2010), distancing may be understood not primarily as indifference, but as a way of enduring the discrepancy between professional ideals and limited practical capacity. In the present findings, such distancing became visible in the use of depersonalised administrative language, through which the child's and family's situation was rendered into cases, categories and documented needs. By speaking in terms of what was verified, legally relevant and permitted within the framework of the law, the bureaucrat could remain within what was institutionally defensible. In this light, hiding behind what the law allows may be understood not only as bureaucratic rigidity but also as a way of bearing the strain of not being able to respond fully to what is at stake in the child's and family's situation.

At the same time, a broader interpretive movement becomes visible. The sustained attention to documentation, legal justification and procedural correctness suggests how the process itself becomes a central horizon of professional being. Through this orientation, the lived reality of the child and family remains largely outside the immediate professional scope. This can be understood as an effect of structural conditions, where acting responsibly requires adherence to procedural forms. In this sense, the findings echo Kafka's portrayal in *The Trial* (Kafka 1925/2009), where necessity derives its authority from the process itself. Formality appears as both protective and distancing, enabling decisions to be justified and shared while also shaping how care can be perceived and enacted.

one doesn't have to take everything as the truth, one just has to accept it as necessary.

(Kafka 1925/2009, 159)

Read through De Leeuw's (2018) interpretation of Ricoeur's ethical aim of ‘the good life, with and for others, in just institutions’ (Ricoeur 1992), the findings may be understood as showing that bureaucratic work is lived in the tension between responsiveness to others and institutional limitation. The bureaucrats appeared not merely as administrators, but as persons trying to act for the child and family within organisational, legal and economic constraints. At the same time, the findings suggest that institutions both enable and constrain such action. They make care possible, yet also call forth distancing and recourse to what the law permits when needs exceed what the system can bear. The study therefore raises the question not only of how children and families are supported but also of how institutions shape both the care made possible and the conditions under which bureaucrats are expected to uphold it. From this perspective, Ray's theory of bureaucratic caring (Ray 2021) offers a further step in understanding the present findings. If caring is inseparable from the legal, economic, political, organisational and technological structures through which care is delivered, an ethos of care cannot rest solely with the individual bureaucrat or carer who stands closest to the child and family. Rather, it must be carried throughout the institutional chain that shapes decisions, resources, responsibilities and possibilities for action. Otherwise, the ethical burden risks being displaced onto those at the front line, who are left to reconcile compassion with legal limits, resource scarcity and organisational demands. In this regard, the present findings also resonate with Altermark's (2025) analysis of how public agencies may seek to manage employees' moral distress when implementing restrictive policy. His study of the Swedish Social Insurance Agency shows how distancing staff from claimants, obscuring individual responsibility, reducing professional autonomy and promoting an organisational ethos of duty can function to suppress employee conscience and facilitate stricter assessments. In light of this, the present findings may be read not only as showing the strain carried by individual bureaucrats but also as pointing to the moral significance of how institutions themselves are organised. If Ricoeur invites the question of whether institutions are just, Ray and Altermark together invite the further question of whether those institutions are organised in ways that sustain caring, or in ways that manage and suppress the conscience of those expected to uphold them.

## 4 Methodological Reflections

To capture a wide range of experiences related to the phenomenon of acting as a street‐level bureaucrat in facilitating home care for children living with respiratory support and their families, participants from a range of professional and organisational roles were recruited. This allowed the phenomenon to be approached from several positions within the welfare system, strengthening the breadth of the material. At the same time, variation in roles and responsibilities made condensation of the material more complex. The material reflected participants positioned both in eligibility assessment and in the organisation and provision of care, which broadened the understanding of the phenomenon but also required careful interpretation across different parts of the process.

Conducting the interviews via Microsoft Teams enabled nationwide participation, including professionals from different parts of the welfare system. This broadened the empirical material, although the digital format may have limited access to workplace context and atmosphere. At the same time, online interviewing may have given participants a greater sense of control and made it easier to speak openly about sensitive matters (Thunberg and Arnell 2022). Access to participants was uneven. In particular, recruiting participants from certain parts of the Swedish Social Insurance Agency proved difficult. Decision‐makers and senior advisory roles were not represented, and some resistance appeared at managerial levels. This limited access has previously been described and should be considered when interpreting the findings (Altermark 2025). Some municipal participants had only limited experience of cases involving children living with respiratory support, in some instances only one or two cases. This reflects the rarity of such cases in smaller municipal catchment areas, and the fact that some cases are later transferred to the Social Insurance Agency. This was a challenge both for recruitment to the study and for the bureaucrats themselves, who were sometimes expected to handle highly complex cases despite limited prior experience.

This interpretation was developed from a caring science perspective, and a caring ethos informed the analysis throughout. The findings should therefore be understood as one possible interpretation of the interview material, rather than a claim to a single truth. However, this is considered one of the best interpretations representing the meanings of acting as a street‐level bureaucrat in facilitating home care for children living with respiratory support and their families.

## 5 Conclusion

Bureaucratic work emerges as more than administration; it is experienced as being stretched between what is called for and what is possible. The findings show how the vulnerability of the individual child and family meets a system shaped by legal categories, organisational routines and economic priorities that at times appear to take precedence over the demands of care. This points to a value conflict between the vulnerability and needs of the individual and the priorities embedded in the welfare system, and raises broader questions about the political responsibility to create just institutions capable of sustaining care in practice. In line with Ray's theory of Bureaucratic Caring, the study suggests that a caring ethos must permeate not only frontline practice but also the organisational and political structures through which care is justified, resourced and delivered. Further research is needed to explore how these organisational and administrative processes are experienced from the perspective of families themselves.

## Author Contributions

Project administration: J.F. Interviews: J.F. Data analysis: J.F. in collaboration with Å.I.‐S., S.O. and B.L. First manuscript draft: J.F. Contribution to study design, critical revision for important intellectual content and approval of the final version to be published: J.F., B.L., S.O., A.M., A.P. and Å.I.‐S.

## Ethics Statement

The study was conducted in accordance with the Declaration of Helsinki [53] and was approved by the Swedish Ethical Review Authority (Dnr 2025‐01875‐01). Written informed consent was obtained from all participants.

## Conflicts of Interest

None of the authors declares any conflict of interest related to this work. Unrelated to this work, A.M. and A.P. have received a grant from ResMed for lectures.

## Declaration of Generative AI and AI‐Assisted Technologies in the Manuscript Preparation Process

Copilot was used for language editing and grammar checking. After using this service, the authors reviewed and edited the content as needed and take full responsibility for the content of the published article.

## Data Availability

The data are not publicly available due to privacy or ethical restrictions.
